# Loss of Expression of a Novel Chromatin Remodeler SMARCA1 in Soft Tissue Sarcoma

**DOI:** 10.4172/2157-7099.1000524

**Published:** 2018-11-23

**Authors:** Pallavi A Patil, Kara Lombardo, Ashlee Sturtevant, Shamlal Mangray, Evgeny Yakirevich

**Affiliations:** Department of Pathology, Warren Alpert Medical School of Brown University, Lifespan Academic Medical Center, Providence, Rhode Island, USA

**Keywords:** Chromatin remodeler, SMARCA1, SNF2L, SWI-SNF, Soft tissue, Sarcoma, Undifferentiated, Leiomyosarcoma, Liposarcoma, Malignant peripheral nerve sheath tumor (MPNST)

## Abstract

**Introduction:**

Vital cellular processes such as proliferation and differentiation are regulated by chromatin remodeling complexes. A variety of neoplasms have been discovered to have genomic alterations (GAs) and loss of immunohistochemical (IHC) expression of chromatin remodelers *ARID1A (BAF250A), SMARCA2 (BRM), SMARCA4 (BRG1),* and *SMARCB1 (INI1). SMARCA1 (SNF2L)* is another member of the chromatin remodelers, and has not yet been studied in neoplasia. As *SMARCA1* is located on chromosome X, could be potentially inactivated by a single hit. We aimed to evaluate GAs and protein expression of *SMARCA1* in soft tissue tumors.

**Method:**

The publically available cBioPortal.32e34 platform was queried to analyze data on soft tissue tumors from The Cancer Genome Atlas project (TCGA) related to *SMARCA1* GAs. Our institutional archives were queried to collect 26 cases of soft tissue tumors including 10 undifferentiated sarcomas, 5 leiomyosarcomas, 6 liposarcomas, and 5 malignant peripheral sheath tumors (MPNST). IHC for SMARCA1 with an SNF 2C4 monoclonal antibody was performed on whole tissue sections.

**Results:**

*SMARCA1* GAs were present in 8/261 soft tissue sarcomas (3%) in the TCGA dataset. Leiomyosarcomas had most common *SMARCA1* GAs in 6/99 cases. *SMARCA1* deletions existed in 1/56 dedifferentiated liposarcomas and 1/48 undifferentiated sarcomas. No *SMARCA1* GAs occurred in other sarcoma subtypes. SMARCA1 IHC was studied in the sarcoma subtypes with potential *SMARCA1* alterations in our institutional cases. SMARCA1 nuclear expression was lost in 3/10 cases (30%) of undifferentiated sarcoma, and 2/5 cases of MPNST (40%). SMARCA1 expression was intact in all cases of leiomyosarcoma and liposarcoma.

**Conclusion:**

This is the first study to demonstrate loss of expression of SMARCA1 in soft tissue sarcomas subtypes, including undifferentiated sarcoma. Our study highlights merit for further investigation on the role of *SMARCA1* in the differentiation process and molecular mechanisms of *SMARCA1* inactivation.

## Introduction

Chromatin remodeling complexes modify the nucleosomal architecture, which provides compact structure and regulates DNA accessibility [1,2]. Vital cellular processes such as proliferation and differentiation are regulated by chromatin remodeling complexes by facilitating reversible opening of chromatin for binding of transcription factors to regions of DNA that in turn cause gene expression and repression [1,2]. The chromatin remodeling complexes are multi-subunit complexes with ATPase that can be grouped into four families - SWI/SNF, ISWI, CHD, INO80, of which the SWI/SNF is most commonly found altered in human cancers [1–4]. A variety of neoplasms have been discovered to have genomic alterations and loss of immunohistochemical (IHC) expression of chromatin remodelers *ARID1A (BAF250A), SMARCA2 (BRM), SMARCA4 (BRG1),* and *SMARCB1 (INI1)* [4]. *SMARCA1 (SNF2L)* is another member of the chromatin remodelers, and has not yet been studied in soft tissue tumors [5]. The location of *SMARCA1* gene on chromosome X makes it susceptible to gene inactivation by the single hit mechanism [5,6]. We aimed to evaluate genomic alterations and protein expression of SMARCA1 in soft tissue tumors.

## Methodology

In order to study genomic alterations we queried the publically available The Cancer Genome Atlas project (TCGA) platform cBioPortal.32e34 for *SMARCA1* in soft tissue tumors to analyze the available data. For performing IHC studies, our institutional records were queried for cases of soft tissue sarcoma diagnosed between 2008 and 2018. Twenty six cases were selected from our institutional archives including 10 undifferentiated sarcomas, 5 leiomyosarcomas, 6 liposarcomas, and 5 malignant peripheral sheath tumors (MPNST). IHC staining was performed on paraffin embedded whole tissue sections on Ventana Discovery with the rat monoclonal antibody SMARCA1 (Anti-SNF2L antibody, clone SNF 2C4, MilliporeSigma, United States) in 1:100 dilution. Normal testis tissue was used as a positive control. SMARCA1 expression was evaluated in tumor cell nuclei. When adjacent normal tissue was present for evaluation, SMARCA1 expression was evaluated in nuclei of adjacent nerve, smooth muscle, adipose tissue, endothelium and stromal cells. SMARCA1 expression on IHC was considered intact when expressed in >5% of nuclei of tissue of interest, and lost when not expressed in >5% of nuclei. Results were tabulated in an IRB approved database.

## Results

The dataset from TCGA contained 261 soft tissue sarcomas. *SMARCA1* genomic alterations were found in eight (3%) of these cases. Leiomyosarcomas were the most common to harbor *SMARCA1* genomic alterations, being found in 6 of 99 cases (6%). Of these deletions were found in 3 cases, missense mutation in 1 case, and amplification in 1 case. *SMARCA1* deletions were present in only one case of dedifferentiated liposarcoma among 56 cases of liposarcomas (1.7%) and in only one of 48 (2%) undifferentiated sarcomas. None of the other sarcoma subtypes had *SMARCA1* genomic alterations.

**Figure 1:**
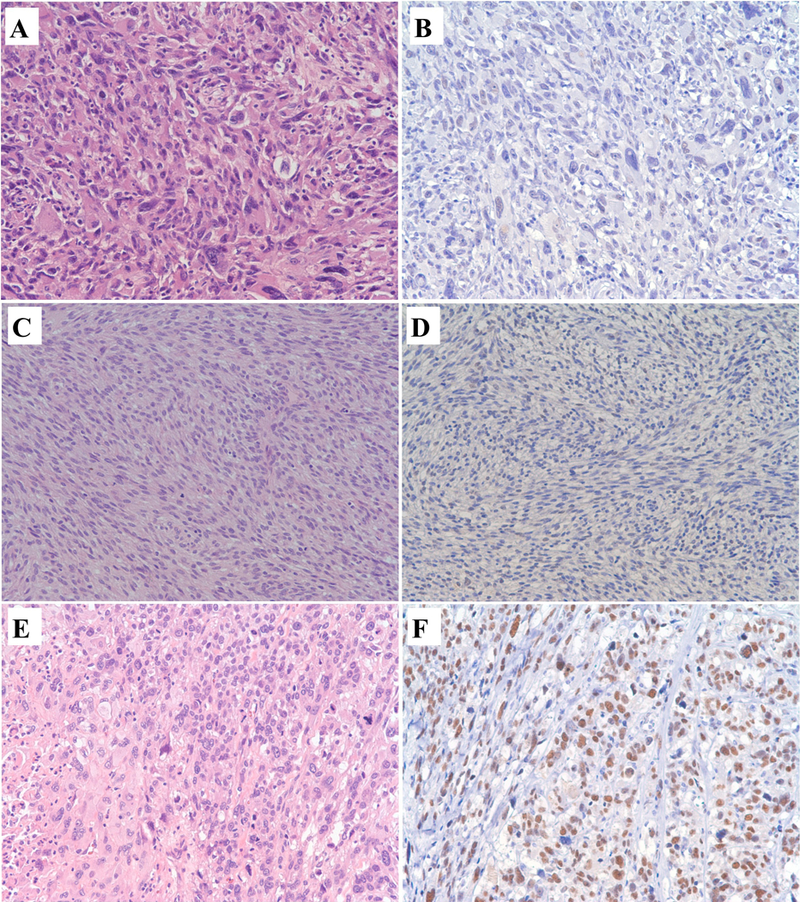
SMARCA1 immunohistochemistry in soft tissue sarcomas, A to F, Light Microscopy, 200x. A and B: Undifferentiated Sarcoma. A, Hematoxylin and Eosin: Undifferentiated sarcoma with haphazardly arranged and vaguely storiform, pleomorphic, anaplastic tumor cells with giant cells and hyperchromatic irregular nuclei. B. SMARCA1 Immunohistochemistry: Loss of expression of SMARCA1 in undifferentiated sarcoma. C and D: Malignant peripheral nerve sheath tumor. C, Hematoxylin and Eosin: Malignant peripheral nerve sheath tumor with tumor cells arranged in sweeping fascicles that are hypercellular, elongated nuclei and mitoses. D. SMARCA1 Immunohistochemistry: Loss of expression of SMARCA1 in malignant peripheral nerve sheath tumor. E and F: Leiomyosarcoma. E, Hematoxylin and Eosin: Leiomyosarcoma with tumor cells arranged in vague fascicles with eosinophilic cytoplasm, necrosis, enlarged hyperchromatic pleomorphic nuclei, and mitoses. F. SMARCA1 Immunohistochemistry: Nuclear expression of SMARCA1 in leiomyosarcoma.

SMARCA1 nuclear expression was lost in 3 of 10 cases (30%) of undifferentiated sarcomas. There was no difference in morphology or nuclear features of tumor cells between the positive and negative cases of undifferentiated sarcoma. Representative images are shown in Figure 1A and 1B. There was loss of expression of SMARCA1 in 2 of 5 cases of MPNST (40%), shown in Figure 1C and 1D. No difference in nuclear features or morphology was noted between the positive and negative cases of MPNST. SMARCA1 expression was intact in all cases of leiomyosarcoma (n=5), shown in Figure 1E and 1F. SMARCA1 was also expressed in all cases of liposarcoma (n=8), dedifferentiated, pleomorphic, and myxoid subtypes.

Expression of SMARCA1 was noted in 56% of adjacent normal smooth muscle, endothelium, and stromal cells. In adjacent normal adipose tissue, SMARCA1 was expressed patchy in only 1 case (5%). In these tissues, the expression was patchy and nuclear. SMARCA1 was not expressed in any of the adjacent normal peripheral nerves 0% (n=8). In the 3 cases of undifferentiated sarcoma with loss of SMARCA1, the adjacent normal tissue also did not express SMARCA1. Of the two cases of liposarcoma and MPNST with SMARCA1 loss, one case each had expression in adjacent normal tissue.

## Discussion

Chromatin remodeling complexes maintain stability and compactness of the DNA through the nucleosomes and regulate accessibility of the DNA to transcription factors [1,2]. The chromatin remodeling complexes can in turn be altered by epigenetic events like methylation, mutations or genomic alterations [5]. The chromatin remodeling complexes contain four families, namely SWI/SNF, ISWI, CHD, INO80 [5]. Among these the ISWI family contains *SNF2L* or *SMARCA1,* and *SNF2H* or *SMARCA5* [4,5,7]. Chromatin remodeling complexes have been found to be altered in a number of tumors types and have diagnostic, potential prognostic and therapeutic purposes [4,8].

*SMARCA2* and *SMARCA4* deficiency was noted in 10% of nonsmall cell lung cancer cases, 80% of *SMARCA2/SMARCA4* deficient tumors was also TTF-1 negative, and recently *SMARCA4* loss was found to be a predictor of response to platinum based chemotherapy [8]. In ovarian cancer, 46% of ovarian clear-cell carcinomas and 30% endometrioid ovarian carcinomas carried *ARID1A* mutations [9]. The loss of BAF250a protein correlated with the tumor subtypes of ovarian clear cell carcinomas and endometrioid carcinoma [9]. Among soft tissue tumors, *SMARCB1* (also known as INI1, hSNF5) has been found altered in malignant extrarenal rhabdoid tumor (mutations), epithelioid sarcoma (deletions), epithelioid malignant peripheral nerve sheath tumor (loss of expression), myoepithelial carcinoma (loss of expression), and extraskeletal myxoid chondrosarcoma (loss of expression) [10,11]. The mechanisms for loss of expression on IHC vary in these tumors, however loss of expression of SMARCB1 (INI1) is useful for diagnostic purposes, and could have potentially therapeutic purposes [11].

Synthetic lethality is co-occurrence of two genetic events that can be used for cell death, and exploited for cancer therapy [12]. In context to chromatic remodeling complexes, some synthetic lethal relationships of SWI/SNF complexes have been described and could be exploited for cancer therapy [1]. The epigenetic mechanism of action of chromatin remodeling complexes renders them to a wide range of effects, making specific targeting for cancer therapy difficult [1]. Extensive research into the role of chromatin remodeling complexes may possibly enable to utilize their potential for therapeutic purposes. The fact that chromatin remodeling complexes are composed of multiple units, loss of one unit can lead to perturbation of function of the complex [1]. Mutations or alterations of the chromatin remodeling complexes also co-occur with other oncogenic alterations, e.g. *KRAS, BRAF* [1].

Our search of literature revealed there are occasional rare studies on *SMARCA1 (SNF2L),* and none on *SMARCA1* in soft tissue sarcoma [4,5]. *SMARCA1* is expressed in a broad range of normal tissues and has been reported to modulate the Wnt/ß catenin pathway [4]. A recent study on gastric carcinomas found *SMARCA1* silenced by aberrant methylation in gastric cancer cells [5].

We found genomic alterations in soft tissue sarcomas in TCGA dataset, namely leiomyosarcomas, liposarcomas and undifferentiated sarcomas. Data on methylation was not available in the dataset. The finding of SMARCA1 IHC loss in undifferentiated sarcoma is in keeping with TCGA molecular alterations. The fact that we did not find SMARCA1 IHC loss of expression in leiomyosarcomas and liposarcomas may be due to the different tumor cohorts and smaller number of cases in our study. In addition, *SMARCA1* genomic alterations may have different effect on protein expression. Loss of SMARCA1 IHC expression in MPNST with no genomic alterations in this subtype suggests that other mechanisms may drive *SMARCA1* down regulation on the transcriptional or posttranslational level. Further investigation into loss of expression of SMARCA1 with more cases is required to study the association between *SMARCA1* genomic alterations and protein expression, and the utility of SMARCA1 loss of expression for diagnostic and prognostic purposes. More studies on regulation and function of *SMARCA1* are required to further understand its role in neoplasia and possibly elucidate interaction with other molecules and chromatin remodeling complexes for better therapy.

## Conclusion

Our study is the first to demonstrate loss of expression of SMARCA1 in subtypes of soft tissue sarcomas, including undifferentiated sarcoma and malignant peripheral nerve sheath tumors. The role of *SMARCA1* needs to be further explored in the differentiation process and neoplasia. The molecular mechanisms of *SMARCA1* inactivation merit further investigation in order to understand and utilize its role in diagnostic, prognostic and therapeutic purposes.
